# Case report of congenital intestinal malrotation in an adult discovered three months status-post appendectomy

**DOI:** 10.1016/j.ijscr.2022.106795

**Published:** 2022-01-24

**Authors:** Logan D. Glosser, Conner V. Lombardi, Hanna M. Knauss, Rachel Rivero, Shirley Liu, Tyler J. Jones

**Affiliations:** a University of Toledo, College of Medicine and Life Sciences, 3000 Arlington Ave, Toledo, OH 43614, USA; b Yale New Haven Hospital, Department of Surgery, 800 Howard Ave # 3, New Haven, CT 06519, USA

**Keywords:** Intestinal malrotation, Volvulus, Ladd's bands, Complications, Case report

## Abstract

**Introduction and importance:**

Intestinal malrotation is a congenital anomaly primarily diagnosed in children, with limited cases reported in adults. Prompt recognition is necessary to prevent life-threatening complications including bowel ischemia and death. We present a rare case of adult intestinal malrotation highlighting difficulty in diagnosis and surgical management.

**Case presentation:**

A 37-year-old Caucasian woman presented with a 3-day history of worsening diffuse abdominal pain, three months status-post laparoscopic appendectomy. CT scan with contrast of the abdomen and pelvis demonstrated small bowel mesenteric swirling and descending duodenal transition point. Differential diagnosis included intestinal malrotation versus small bowel obstruction. Pre-operatively, the patient expressed frustration with years of abdominal pain and lack of improvement. Treatment with open surgical small bowel detorsion and ligation of the Ladd's bands was performed, after initial laparoscopic intervention was complicated by enterotomy. The patient recovered well post-operatively with final diagnosis of intestinal malrotation with midgut volvulus. Discharge home was delayed due to polysubstance withdrawal. Post-operatively, the patient reported immediate relief of symptoms which persisted at 2-week and 2-month follow-ups.

**Clinical discussion:**

Few reports of congenital malrotation diagnosed in adulthood are reported. This highlights the importance of evaluating all patients for malrotation when the appendix is found outside of the normal positioning in the RLQ, as surgical correction of malrotation is of utmost importance in such patients.

**Conclusion:**

Clinicians should consider intestinal malrotation in adults with recurrent vague abdominal symptoms. To our knowledge, this is the first report of congenital malrotation discovered in an adult after prior appendectomy.

## Highlights

• Congenital intestinal malrotation diagnosed in adults is scarcely reported.
• No guidelines exist for the management of malrotation in adult patients.
• To our knowledge, this is the first report of intestinal malrotation discovered after previous appendectomy.

## 1 Introduction

Intestinal malrotation is a congenital anomaly predominantly diagnosed in the first year of life, with only 0.2–0.5% of cases reported in adolescents and adults [1]. Malrotation is associated with significant morbidity, thus diagnostic criteria, understanding of anatomy, and prompt intervention is imperative for optimizing outcomes [2]. Intestinal malrotation is defined by any deviation from the normal 270 degree counterclockwise turn of the midgut and has been described as “a time bomb lying within” due to consequential intestinal necrosis without prompt surgical detorsion [3], [4].

In adults, most patients are asymptomatic with incidental discovery during surgical intervention for other disease management. However, some adults may present with vague abdominal complaints, symptoms of chronic intermittent obstruction, or rarely as an acute abdomen [5]. Often the symptoms in adults are mistaken for other diseases including peptic ulcer disease, gastro-esophageal reflux disease, irritable bowel syndrome, biliary or pancreatic disease, and psychiatric disorders [6]. Due to scarcity of adult cases, timing and approach to surgical management is not well defined without specific guidelines [5], [7]. Specifically, there are no management guidelines for intervention if malrotation is incidentally discovered during another operation. The management of this patient was at an academic university-based hospital. This case report has been reported in line with the SCARE Criteria [8].

## 2 Presentation of case

A 37-year-old Caucasian woman presented to the emergency department, walked in after being brought by a friend, with three days of worsening diffuse sharp abdominal pain associated with nausea, vomiting, and chills. Patient reported her last bowel movement was a few days prior with minimal flatus, denying presence of diarrhea, melena, hematochezia, or fever. Past medical history included chronic abdominal pain, chronic biliary duct dilatation, polysubstance abuse (cocaine, heroin, marijuana, and alcohol) on 110 mg oral methadone therapy, anxiety, depression, PTSD, recurrent colitis, and ruptured appendicitis. The patient was also a victim of sexual and domestic assault and had attempted suicide multiple times. She had no known allergies. Social history was significant for current tobacco use with 8.5 pack year smoking history, near-daily alcohol consumption and unemployed.

**Fig. 1:**
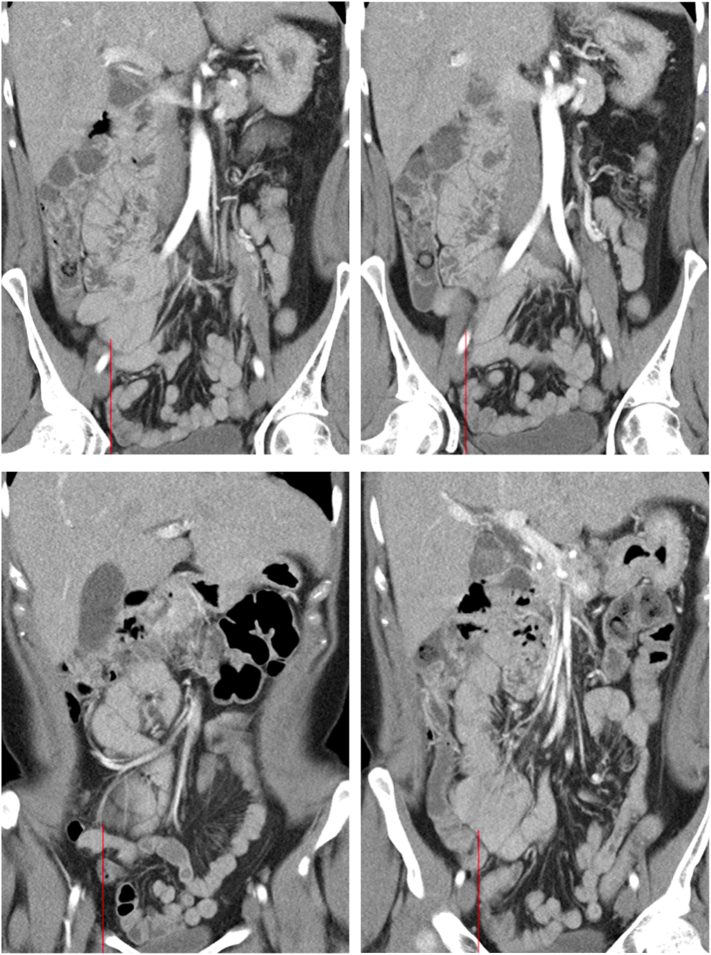
CT images from prior ED visit 2 months before hospital admission for abdominal pain with no mention of malrotation.

The patient underwent laparoscopic appendectomy three months prior complicated by post-operative abscess requiring IR drainage. Intraoperatively, the cecum, terminal ileal loops, and appendix were noted to be attached in an inflammatory mass. The cecum was adhered to the lateral abdominal wall and the appendix was curled unto itself obscured by inflammatory tissue. Notably, neither the operative report nor CT scans during the encounter identified malrotation (Fig. 1), however a dilated CBD duct was repeatedly noted likely related to chronic methadone therapy. After the appendectomy, the patient returned to the ED three times for non-specific abdominal pain. Repeat CT of abdomen and pelvis noted improvement of the peri-appendiceal abscess with incidental finding of malrotation, which was subsequently seen on comparison of prior CT images.

**Fig. 2:**
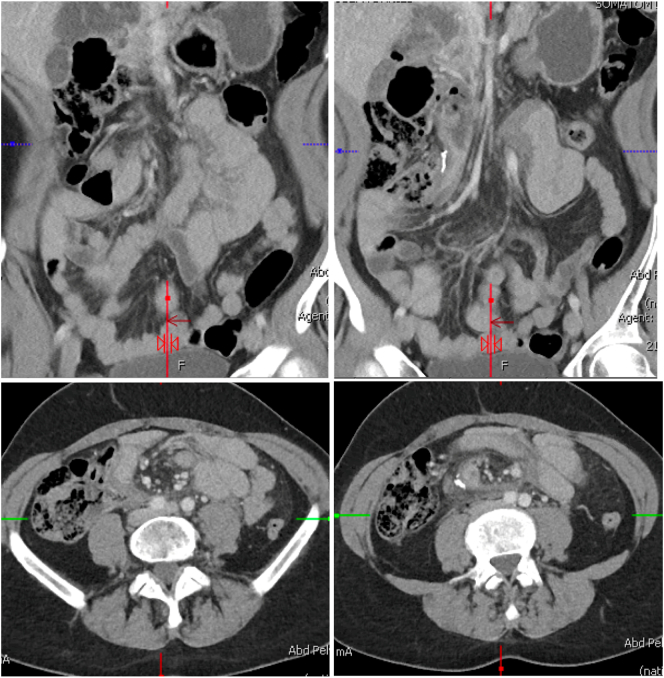
CT images from ED on hospital admission day 1 showing mesenteric swirling, SBO, and possible midgut volvulus.

Upon examination, the patient reported same-day cocaine use prior to the early morning ED arrival. Vital signs and laboratory analyses were within normal limits (WNL). Physical exam was notable for signs of acute abdomen with rebound tenderness and guarding. CT images were concerning for closed loop SBO located at the proximal small bowel/duodenum and the staple line of her prior appendectomy with a transition point at the level of the second portion of the duodenum (Fig. 2). Other findings included mesenteric edema and swirling, possibly related to adhesions or volvulus, and incidental discovery of a Phrygian cap on the gallbladder. Additionally, high grade stenosis of 2 superior mesenteric veins was seen, however the superior and inferior portions of the branches remained patent. The differential diagnosis included mesenteric volvulus vs. internal hernia.

After discussing with the patient risks, benefits, and alternatives of diagnostic laparoscopy, possible exploratory laparotomy, and possible bowel resection, decision was made to proceed with surgical intervention. The patient was eventually taken to the OR under the care of a junior faculty general surgeon and placed under general anesthesia. Supra-umbilical local anesthetic was injected and a 12 mm vertical incision was made. The fascia was grasped and elevated with Kochers and divided vertically in between. The posterior sheath was similarly grasped, elevated, and cut. A finger sweep revealed another layer consistent with peritoneum, so this was grasped, elevated, and cut. At this point a final finger sweep was performed which returned feculent matter. On further inspection an incidental colotomy was grossly visible, thus the decision was made to convert to an open exploratory laparotomy.

**Fig. 3:**
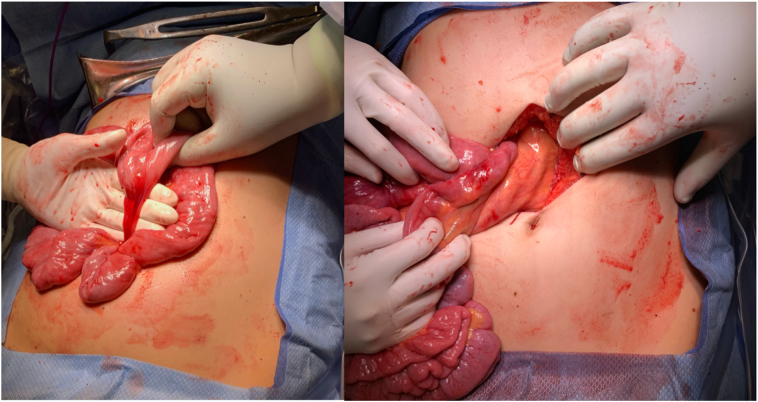
Intraoperative visualization of the midgut volvulus with twisting of the mesentery around one of the fibrous bands.

**Fig. 4:**
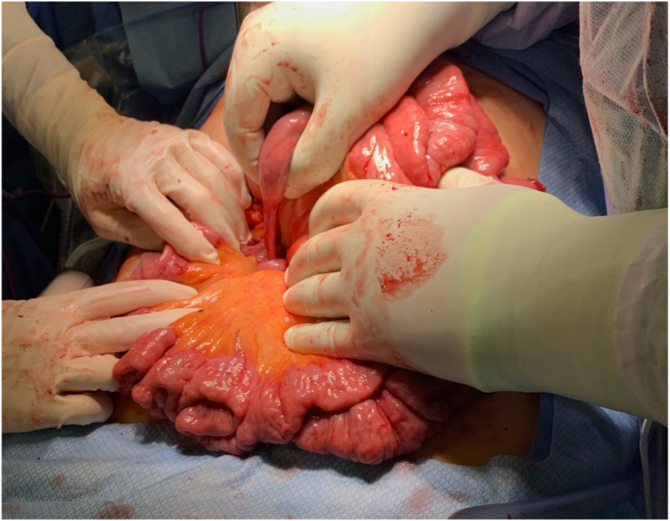
Ladd's bands connecting the small bowel to the anterior abdominal wall.

An upper midline incision was made and carried through the fascia into the peritoneal cavity. The colon was eviscerated and the colotomy site was identified in the distal colon. There was no spillage and edges were healthy, so the site was closed and oversewn with silk sutures in Lembert fashion. Attention was then turned to the small bowel which appeared completely volvulized (Fig. 3). Prior to detorsing the bowel, six Ladd's bands were carefully identified to ensure no mesenteric injury. The bands were then divided as they were fixating the proximal bowel to the root of the mesentery and antero-lateral abdominal wall (Fig. 4). One of these Ladd's bands was intimately associated with the cecal staple line from the prior appendectomy. After dividing the Ladd's bands the small bowel was able to be completely detorsed by rotating it around its mesenteric axis in a counterclockwise fashion. The bowel was investigated twice from the ligament of Treitz to the cecum and without evidence of ischemia, perforation, or mesenteric injury. The mesentery was narrow with portions of bowel attached to a “stalk.”

To broaden the mesentery, peritoneal attachments of the right colon to the right upper quadrant (RUQ) abdominal wall were divided using electrocautery and the gastrocolic ligament was partially divided on the right side to allow the colon to be medialized and swept away from the duodenum. The mesentery subsequently appeared adequately broadened. No further division of the gastrocolic ligament or peritoneum overlying the actual mesenteric vessels was performed, as it risked mesenteric injury. After placing the colon to the left of the peritoneal cavity, the small bowel was returned to the right side. The abdomen was irrigated and bowel reinspected which showed viability and intact colotomy repair. The fascia and skin were closed, and the patient had an uneventful emergence from anesthesia. A nasogastric (NG) tube to suction was placed before extubating.

DVT/VTE prophylaxis was initiated pre-operatively with enoxaparin 40 mg injections daily. Pain was controlled with acetaminophen 1000 mg IV q8 and morphine 4 mg IV q6 PRN. Oral pain medications were not administered to protect bowel function. The NG tube was removed on POD-2 at which the patient was successfully transitioned from NPO to full diet by POD-3. The patient tolerated the procedure well without any surgery-related adverse events; however, the post-operative course was complicated by poly-substance abuse withdrawal. The withdrawal symptoms were successfully managed with the clinical institute withdrawal Assessment (CIWA) protocol. The patient was discharged on hospital day 8 with continued methadone therapy and instructed to take over the counter acetaminophen as needed. At the 2-week appointment the patient stated she did not require use of the acetaminophen and confirmed relief of symptoms at 2-week and 2-month in person postoperative follow-up appointments, without residual abdominal pain. The patient self-reported adherence to 2-week avoidance of heavy lifting >20 pounds. The patient self-reported tolerating a normal adult diet without restriction and regular bowel movements every other day. At this point the patient did not require any future follow-up appointments. No post-discharge labs were indicated nor performed.

## 3 Discussion

Intestinal malrotation is a congenital anomaly with 90% of cases diagnosed in the first year of life [4]. Adult presentation is rare, with an estimated incidence of 0.2% [4]. Most cases are found incidentally on imaging or during surgical intervention for other comorbidities [5]. The index of suspicion for congenital malrotation decreases with age, and diagnosis is often missed on initial evaluation [9].

In normal embryonic intestinal rotation, the midgut herniates through the umbilical cord between 4 and 6 weeks gestation as it outgrows the abdominal cavity and returns by the 10th week [4], [10]. The midgut receives blood supply through the superior mesenteric artery (SMA) and rotates 270 degrees counter-clockwise from herniation to peritoneal return [4]. The rotation leads to ultimate orientation with the duodenal-jejunal flexure on the left side and descent of the cecum into the right lower quadrant, anchored to the abdominal wall by fibrous tissue known as Ladd's bands [4], [10]. The ascending colon assumes retroperitoneal position on the right side and the small bowel mesentery anchors to the peritoneum through the ligament of Treitz at the duodenal-jejunal flexure [10].

Typically in malrotation the duodenal-jejunal flexure orients on the right side with absence of peritoneal fixation through the ligament of Treitz [4], [10]. The cecum, appendix, and colon lie on the left side, however, the Ladd's bands remain attached to the cecum and the right abdominal wall, posing risk for entrapment and obstruction of the small bowel [4], [11]. The resulting anomaly lacks peritoneal fixation, making the small bowel mobile and susceptible to subsequent volvulus [11].

In children, malrotation commonly presents with bilious vomiting, abdominal distension, hematochezia, or failure to thrive [10]. Adult presentation is non-specific and ranges from acute to chronic complaints. Symptoms of chronic malrotation and volvulus include constipation, nausea, vomiting, and intermittent abdominal pain [4]. This is believed to be due in part to compression of the small bowel by Ladd's bands [4]. Our patient had three ED visits for right-sided colicky abdominal pain in the three months prior and then presented with signs of acute abdomen with rebound tenderness and guarding. Acute presentation is typically characterized by symptoms of small bowel obstruction, peritonitis, or appendicitis [4]. Due to disruption of normal positioning of the appendix that may result from malrotation anomalies, the clinical presentation may not present in the right lower quadrant, as in our patient in which the appendix was found to be stuck to the cecum and terminal ileum in the RUQ. Presentation of other abdominal conditions may further be obscured by the abnormal anatomy.

Diagnosis of intestinal malrotation in adults is difficult due to the vague nature of symptoms, scarcity of reported cases, and diagnostic recommendations limited to pediatric populations. In children, upper GI series are the diagnostic modality of choice, however, prior case reports and series demonstrate a lack of sensitivity in adults, especially those who present with more distal obstruction [5], [7], [11]. One review found the diagnosis was made on imaging in 5 of 12 adults who all underwent upper gastrointestinal contrast study, barium enema, or CT scan [5]. The remaining 7 cases were not identified on preoperative studies and discovered incidentally during surgery [5]. To increase sensitivity, an upper GI series combined with barium enema is the recommended gold standard for diagnosis [5].

In our patient, malrotation was identified on a CT scan six months prior to presentation with the appendicitis, however it was not reported on four CT scans prior to surgical intervention. To prevent diagnostic delay, radiologists should be made aware of the twisted corkscrew appearance of the duodenum on upper GI contrast studies, the “swirl” or “whirlpool” sign due to the bowel and mesentery encircling the SMA on CT, and a “beak” appearance of intestinal stenosis associated with volvulus on barium enema [2], [5], [7], [11]. In our patient, CT scan showed likely congenital small bowel malrotation with jejunal loops in the RUQ with an acute proximal SBO with a transition point at the second portion of the duodenum near the appendectomy stable line. Additionally, the small bowel mesentery showed a swirled appearance concerning for possible mesenteric volvulus given the malrotation variant morphology. Other modalities used to diagnose malrotation include magnetic resonance imaging (MRI), ultrasound, and arteriography [9], [12].

The Ladd procedure is the surgical intervention for intestinal malrotation encompassing volvulus detorsion, Ladd's bands ligation, broadening the mesenteric base, and appendectomy [5], [7]. Open surgical intervention is generally preferred over laparoscopic, due to increased physician comfort and less risk of incomplete procedure, particularly when accessing posterior duodenal attachments [5], [7]. In the case of our patient, an incidental enterotomy was made during the Hassan technique for initial peritoneal access, thus decision was then made to convert to open for enterotomy repair, ligation of Ladd's bands, and manual detorsion of the midgut volvulus. As the patient had already received an appendectomy 3 months prior, this portion of the Ladd's procedure was not required.

## 4 Conclusion

Intestinal malrotation is a rare cause of acute abdomen in adults because of congenital disruption of bowel rotation during fetal development. Currently, no standard of care exists for the surgical approach to intestinal malrotation in adults. Our case adds a new perspective to the literature as no prior cases of malrotation discovery after surgical appendectomy exist and we bring awareness to this rare disease as this patient may have benefitted from the Ladd's procedure during the initial appendectomy, as this may have prevented the second operation.

## Sources of funding

This research did not receive any specific grant from funding agencies in the public, commercial, or not-for-profit sectors.

## Ethical approval

N/a.

## Consent

Written informed consent was obtained from the patient for publication of this case report and accompanying images. A copy of the written consent is available for review by the Editor-in-Chief of this journal on request.

## Provenance and peer review

Not commissioned, externally peer-reviewed.

## Registration of research studies

N/A.

## Guarantor

Logan D. Glosser.

## CRediT authorship contribution statement

All authors read and approved the final version to be published.

Logan D. Glosser: Investigation, Writing – Original Draft, Writing – Review & Editing, Project administration.

Conner Lombardi: Investigation, Writing – Original Draft, Visualization.

Hanna Knauss: Writing – Review & editing.

Rachel Rivero: Validation.

Shirley Liu: Supervision

Tyler Jones: Conceptualization.

## Declaration of competing interest

The authors have no conflicts of interest to declare.
